# Amino acid restriction, aging, and longevity: an update

**DOI:** 10.3389/fragi.2024.1393216

**Published:** 2024-05-02

**Authors:** S. N. Austad, J. R. Smith, J. M. Hoffman

**Affiliations:** ^1^ Department of Biology, University of Alabama at Birmingham, Birmingham, AL, United States; ^2^ Department of Biological Sciences, Augusta University, Augusta, GA, United States

**Keywords:** longevity, diet, food restriction, protein restriction, amino acid restriction

## Abstract

Various so-called dietary restriction paradigms have shown promise for extending health and life. All such paradigms rely on *ad libitum* (hereafter *ad lib*) feeding, something virtually never employed in animals whose long-term health we value, either as a control or, except for food restriction itself, for both control and treatment arms of the experiment. Even though the mechanism(s) remain only vaguely understood, compared to *ad lib*-fed animals a host of dietary manipulations, including calorie restriction, low protein, methionine, branched-chain amino acids, and even low isoleucine have demonstrable health benefits in laboratory species in a standard laboratory environment. The remaining challenge is to determine whether these health benefits remain in more realistic environments and how they interact with other health enhancing treatments such as exercise or emerging geroprotective drugs. Here we review the current state of the field of amino acid restriction on longevity of animal models and evaluate its translational potential.

## A brief history of dietary restriction

Ever since the discovery that restricting laboratory rodent food consumption relative to their *ad libitum* (henceforth *ad lib*) feeding amount reliably extended their lives (McCay et al., 1935), prevented or delayed a host of diseases, and generally enhanced later life health (Weindruch and Walford, 1988), researchers have been seeking to discover the mechanism(s) by which such restriction works. One way to investigate this question is to determine whether a key feature of what we call the dietary restriction (DR) effect, that is, improved health, reduced disease and extended longevity due to diminished food consumption, is to restrict various components of the diet as contrasted with simply reducing food consumption itself. By now, experimental reduction of all dietary macronutrients has been performed many times in addition to macronutrient components, particularly essential amino acids, in multiple species.

Several aspects of the DR effect are striking. First, with some exceptions (Carey et al., 2002; Liao et al., 2010; Schleit et al., 2013), it seemed to “work,” *i.e.*, extend longevity, across a wide range of genotypes and species including yeast, rotifers, nematodes, water fleas, spiders, fruit flies, guppies, mice, rats, hamsters (Weindruch and Walford, 1988; Mair et al., 2009), and under some circumstances primates (Mattison et al., 2012; Colman et al., 2014). Early researchers sought insight by investigating such questions as how much food intake could be reduced while still observing extended longevity, and how the duration of restriction, or age at which it was initiated, affected both longevity and later life pathology (Weindruch and Walford, 1988). In laboratory rodents, food restriction by as much as 65% (Weindruch et al., 1986) or as little as 10% (Richardson et al., 2016) was found to extend life. Severe restriction, however, can have side effects including loss of muscle mass and bone mineral density (Villareal et al., 2006) as well as increasing susceptibility to death from pathogenic infections (Sun et al., 2001; Kristan, 2008; Ritz et al., 2008; Ayres and Schneider, 2009), something that is often overlooked in touting its benefits. The received wisdom in the field evolved to conclude that the impact of DR on survival increased the longer its duration, the greater the restriction level (short of starvation) and the earlier in life that it was initiated as the largest effects in laboratory rodents were found when DR began soon after weaning when animals were only partially grown (Weindruch and Walford, 1988).

### Laboratory diets

It is worth considering that *ad lib* feeding, which is standard in the husbandry of laboratory rodents, indeed in virtually all laboratory animals, is rare in the husbandry of animals whose long-term health we value. That is, for companion or zoo animals *ad lib* feeding is virtually unheard of except in house cats where it is common practice despite being associated with overweight, obesity, and their attendant health problems (Nelson et al., 1990; Russell et al., 2000; Laflamme, 2005), consequently is discouraged by veterinarians (e.g., https://vcahospitals.com/know-your-pet/feeding-times-and-frequency-for-cats; https://www.ethosvet.com/blog-post/best-practices-for-feeding-your-cat/) and even pet food companies (https://www.hillspet.com/cat-care/nutrition-feeding/how-to-feed-a-cat?; https://www.purina.com/articles/cat/feeding/guides/free-feeding). However *ad lib* feeding is common in commercial production species such as poultry, fish, and pigs, in which the goal is to maximize early life growth and/or reproduction (Leeson and Summers, 2005) and long-term health is considered irrelevant (Hoffman and Valencak, 2020). In fact, commercial diets in laboratory rodents are specifically designed to maximum early life growth and/or reproduction (Lee et al., 2023) for commercial advantage. Not surprisingly then, feeding a fixed ration typically improves health and longevity relative to *ad lib* feeding (Mitchell et al., 2019). In fact, various forms of so-called restriction from *ad lib* feeding all tend to somewhat retard development, growth, and/or reduce reproduction depending on when in life it is initiated. From a perspective that standard rodent diets are formulated to maximize growth rate, it is perhaps less surprising that restricting food intake improves health and increases longevity compared to those with infinite access to food, no opportunity to reproduce, and little opportunity for physical activity as is the case for laboratory animals. One finding that was surprising in the classic food restriction paradigm is that re-feeding later in life typically restores reproduction at ages beyond which *ad lib* fed animals had long since ceased reproducing (Holehan and Merry, 1985a; Holehan and Merry, 1985b).

It has been argued that *ad lib* feeding is the most appropriate preclinical paradigm as humans themselves are *ad lib* fed. We would challenge that humans and laboratory animals cases are comparable. Even setting aside the substantial fraction of the world’s population that lives in chronic food shortage, the human equivalent of a mouse’s *ad lib* feeding would seem to be more like prisoners in a cell with little to do, minimal opportunity for physical activity, and an infinite supply of food within easy arm’s reach. In our opinion, the normal human condition has little resemblance to that scenario.

*Ad lib* feeding, as contrasted with feeding a fixed ration, also has several disadvantages from an experimental perspective. First, there is no control over individual variation in the amount eaten—and this can matter. For instance, variation in individual body weight has been found to account for over 50% of the variation in liver tumor incidence among B6C3F1 mice (Turturro et al., 1996). Second, the actual amount eaten will depend on a host of environmental factors (*e.g.*, number of mice per cate, temperature, humidity, photoperiod) as well as on the palatability and nutrient composition of the diet. For instance, *ad lib* fed mice housed alone ate 40% more food than group-housed mice with no difference in body weight (Ikeno et al., 2005). Also, mice maintained at 22°C ate 30% more than *ad lib* fed mice housed at 27°C with no difference in body weight or composition (Huffman et al., 2007). Another example, animals with continuous access to low protein to carbohydrate ratio (P:C) diets typically eat less than animals on higher P:C diets (Weindruch and Walford, 1988). Relying on *ad lib* feeding also confounds drug testing if the drug is administered in food and affects the amount eaten (Hart et al., 1992; Ellacott et al., 2010). Is the effect from the drug or the DR effect from lower food consumption? Even if that is not the case, individuals will differ in the received drug dose to the extent that they differ in amount they eat. Consequently some DR paradigms feed control animals somewhat less than *ad lib*-fed animals eat (Weindruch and Walford, 1988).

The influence of macronutrient composition of the diet also made a reasonable target of early research as growth retardation was thought to be a key feature of the rodent DR effect. Suspicion arose that reduced protein intake, rather than total caloric, intake might account for the DR effect, because of protein’s known impact on growth rate. Although there were some inconsistencies in the early results, a consensus eventually emerged that although protein restriction properly applied *did* lengthen laboratory rodent life, reduced caloric intake was the main contributor to the DR effect (Masoro et al., 1989; Speakman et al., 2016). In fact, for the past several decades dietary restriction has typically been referred to as “calorie” restriction.

In the 1990s and 2000s as invertebrate models became more heavily used in aging research, virtually every aspect of the conventional wisdom about DR was challenged. Before going into these challenges, we should note that DR is *usually* performed substantially differently in invertebrates than in rodents. Restricted rodents simply have limited food availability. Invertebrate DR typically involves dietary dilution. That is, animals are given less food per unit volume but with no restriction on how much they can consume—*ad lib* feeding in both experimental arms, in other words. In flies specifically, reduced protein, in the form of reduced yeast in *Drosophila* diets, rather than total calories, was claimed to be the main driver of the DR effect (Bass et al., 2007), although other researchers concluded that the key feature was protein:carbohydrate ratio (Lee et al., 2008; Ja et al., 2009). In the early days, food consumption in invertebrates was rarely measured. When it was, some fly researchers presented evidence that flies ate less of the low yeast diets, hence the claimed impact of protein restriction was at least partially due to calorie restriction too (Tatar et al., 2014). Fly DR was also complicated by the impact of hydration on longevity. Some, but not all, researchers found that when flies were given access to free water, the DR effect disappeared (Carey et al., 2002; Ja et al., 2009). Because the impact of protein restriction seemed so striking in flies, and was less well-established in rodents, the idea arose that the DR effect in mammals versus invertebrates might work through different mechanisms (Speakman et al., 2016). In fact, this had been hinted at for some time as laboratory rodent DR was largely thought to involve altered signaling through the insulin/IGF signaling (IIS) network, whereas DR worked as well in laboratory invertebrates even with the IIS network genetically inhibited (Mair et al., 2009). Determining the extent to which mechanisms of various forms of food or nutrient restriction overlap between invertebrates and mammals (or even among species within these groups) remains one of the challenges of understanding nutritional interventions.

In addition, it was discovered that chronic, long-term restriction was not required to see a survival effect. Food restriction produced an almost an immediate survival impact in standard laboratory invertebrates (Mair et al., 2003; Mair et al., 2005). This was later confirmed in laboratory mice as well, in that short-term fasting enhanced their resilience to surgical reperfusion and cancer chemotherapeutic stress (Raffaghello et al., 2008; Mitchell et al., 2010). A period of reduced food, even when mice were still nursing from their mothers was sufficient to provide a life-long survival benefit (Sun et al., 2009). These and other discoveries led to a wealth of new short-term fasting diets including a number of intermittent fasting and time-restricted eating diets which focus on fasting physiology (Austad and Hoffman, 2021). A considerable advantage of these new generation short-term fasting diets is that people can adhere to them in ways that they cannot with chronic calorie restriction (Kraus et al., 2019).

Here it becomes worth briefly exploring what is meant by “restriction” in laboratory dietary studies, particularly as we begin to consider reduction of specific nutrients rather than only the amount of food eaten. Interestingly, the meaning of “restriction” varies depending on the species under study. In mammals, we generally mean either restriction of food availability (DR) relative to animals with unlimited access to food or we mean restriction of specific macro- or micro-nutrients relative to that in the control diet, but both “restricted” and control animals are *ad lib* fed. This brings up the important issue of composition of control diets. Recall that standard laboratory rodent diets are nutritionally designed to maximize growth in early life rather than to maximize health (Lee et al., 2023). Diets designed for other ends, such as reproductive productivity, differ from those used for growth or maintenance (Turturro et al., 1999).

Commercial rodent diets are often assumed to be relatively standardized. They are not. A recent analysis of a range of commonly used rodent diets found protein (as percent of kilocalories in the diet) ranged from 13% to 27%, fat from 3% to 22%, and carbohydrate from 40% to 68% (Lee et al., 2023). As a consequence, it is difficult to identify exactly what nutrient restriction means across studies. Restriction compared to what? In fact dietary manipulations of macro- or micro-nutrients could more accurately called dietary optimization (for survival? for reproduction?) studies rather than nutrient restriction studies. The somewhat arbitrary meaning of restriction has been particularly challenged by the nutritional geometry approach in which macronutrients are systematically manipulated together and the focus is on nutrient ratios rather than absolute amounts (Raubenheimer and Simpson, 2016).

Standardized diets can also be problematic in worms and flies. As we have noted, in laboratory invertebrates, DR typically means dilution of food but no restriction on amount consumed. Food consumption itself is rarely measured, the assumption being that approximately the same volume of diluted food will be consumed as the control diet, something which seems to be largely true for worms (Mair et al., 2009) but not for flies (Carvalho et al., 2005). Notably in laboratory rodents, food dilution with a nondigestible filler does not appear to have the same impact on longevity as does DR (Solon-Biet et al., 2014; Speakman et al., 2016), which appears to be because a period of daily fasting is a critical part of the rodent DR effect (Pak et al., 2021). This difference should be kept in mind when comparing results among taxa. However, the shorter life and larger number of individuals in worm and fly studies allows a broad range of dietary dilutions to be assessed. Interestingly, the composition of control diets in flies is even less standardized that control rodent diets. By comparison, the standard worm diet is relatively standardized in composition (live *E. coli* B strain OP50), but the diet itself is not optimized for any life history trait and in fact is somewhat toxic. Other bacterial species can increase development, reproduction, and longevity (Garsin et al., 2003; Stuhr and Curran, 2020). However, carefully thought-out methods for implementing worm and fly DR based on control diets that maximize fecundity are available for both species (Bass et al., 2007; Mair et al., 2009).

Together this history suggests that in most cases there is an arbitrary aspect to what we call nutrient restriction that seems underappreciated. “Restricted” relative to a control diet designed to maximize growth of young animals, as in laboratory rodents, without concern for long-term health could just as easily be thought of as an “overabundance” of nutrients or calories in the control diet and the experiments called dietary optimization studies instead of “restriction” studies.

### Amino acid restriction

In addition to the impact of total calorie intake and macronutrient composition on health and longevity, the essential amino acid composition of the protein component also matters and has been the object of considerable interest (Grandison et al., 2009). This should have been evident from early on when it was discovered that a life-shortening nephropathy in male F344 rats could be alleviated, and longevity increased, simply by changing the protein source in the semi-purified diet from casein to soy (Weindruch and Masoro, 1991). Soy has a lower percent of aging-relevant amino acids, methionine and branched-chain amino acids, than casein (Luiking et al., 2005). It is also worth noting that excess amino acids or large imbalances in the relative abundance of amino acids have long been known to have toxic side effects, and among the amino acids, methionine has been called the most toxic if provided in excess (Harper et al., 1970).

Three types of amino acid optimization studies have been conducted.

#### Tryptophan

The first single amino acid intervention to be reported to improve and extend rodent health was tryptophan restriction (TR), called tryptophan deficiency by the original authors (Segall and Timiras, 1976). Although the tryptophan metabolism network has some interesting intermediates and end-products from the perspective of aging biology, such as the neurotransmitters serotonin and melatonin as well as NAD, a key player in many aspects of cellular energetics (Hoffman et al., 2019a), TR was first implemented as a less labor-intensive method of DR, because tryptophan deficiency was known to reduce appetite and retard growth in rodents (Segall and Timiras, 1976). However a close examination of the history of TR leads to questions about it having any beneficial effect on health or longevity independent of its impact on food consumption.

Failure to account for reduced food consumption in interpreting dietary as well as pharmacological interventions was a common problem in the 1970s and 1980s and continues to some extent today (Miller et al., 2007). In the initial report, the main positive effect of TR is that returning female Long-Evans rats to a control diet late in life restored reproduction at ages when controls were all postreproductive just as with DR. Better survival was reported only from age 23.5 months when the restricted animals were returned to a control diet for the rest of their lives, but survival up to 23.5 months was considerably worse when TR had been initiated at 3 weeks of age before they were fully grown (Segall and Timiras, 1976). A later paper clearly showed that TR rats actually survived dramatically worse than controls up to age 2 years after which survival favored the remaining restricted animals (Ooka et al., 1988). An independent study of male Swiss albino mice on a relatively high 26% protein control diet found a small survival advantage of animals that were tryptophan restricted from 4 weeks of age. The restricted mice also weighed about 30% less than controls (De Marte and Enesco, 1986). Although it is stated that there was no difference in food consumption between the groups, no data are presented to support that claim. So the evidence that tryptophan deficiency has robust health benefits in laboratory rodents is weak at best. However, detailed modulation of dietary tryptophan has never been performed, so it could be that the optimal or even near optimal dietary tryptophan concentration for survival has not yet been discovered.

#### Methionine (rodents)

The most thoroughly studied single amino acid reduction paradigm has been so-called methionine restriction (MR) and the earliest studies were done using only male rats, a common practice at the time. Methionine has long been known to play a key role in growth and reproduction (Navik et al., 2021) and the earliest study of MR, like that of TR, was due to its known growth-retarding effect (Orentreich et al., 1993) under the assumption that growth retardation was a key feature of DR. It is important to note at the outset that it is well-established that too much methionine is toxic, with adverse effects on appetite, growth, fertility, and multiple aspects of sulfur metabolism (Lee et al., 2014).

The first paper on this topic did not call it MR, but a “low methionine diet,” a better descriptive term. They restricted methionine to 20% of that in the purified control diet (0.17% vs. 0.86% methionine by weight) which was based on a commonly used rodent chow (AIN-76). That level of MR arrested weight gain in these 42 day old, still growing, male F344 rats. Reducing methionine further -- to 0.12% -- resulted in all animals quickly dying (Orentreich et al., 1993). The chemically-defined diets used in this experiment did not contain the other sulfur-containing amino acid, cysteine, for which methionine is a precursor. Therefore cysteine would have also been reduced compared with the control diet. Over the next 50 weeks the MR animals gained no weight whereas the control rats gained 350 g, making the controls about four times the weight of the restricted animals. Yet the stunted MR animals had a median longevity 29% longer than the controls and maximum longevity was also significantly extended. Wisely, these authors periodically measured food consumption, finding that the MR animals ate 10% less than controls during the first 2 months on the diet, 12% less after 3 months, and 24.5% less at age 2 years. Animals pair-fed the control diet at the amount consumed by the low methionine diet showed a growth trajectory similar to the *ad lib*-fed controls, so the growth retardation could clearly be attributed to low methionine. Further work by the Orentreich lab found that low methionine diets extended longevity along with retarding growth in several other genotypes of male rats in addition to the F344 strain (Zimmerman et al., 2003).

In a follow-up publication using a similar experimental protocol to the original study, the low methionine diet this time increased median longevity by 43% and also resulted in animals that weighed 43% less (Richie et al., 1994). The focus of this study was on a mechanism, specifically assessing whether a low methionine diet reduced oxidative stress by inducing the potent antioxidant glutathione as oxidative stress in the 1990s and early 2000s was a favorite putative mechanism of aging. The logic of this study was that the DR effect had repeatedly been shown to correlate with reduced production of reactive oxygen species, often with enhanced cellular antioxidants and reduced oxidative damage to proteins, lipids, and DNA (Bokov et al., 2004). DR animals were also known to be more resistant to induced oxidative stress and genetically increased longevity was associated with enhanced resistance to oxidative stress and less oxidative damage to tissues (Martin et al., 1996; Pamplona and Barja, 2006; Benedetti et al., 2008). Finally, glutathione (GSH:GSSH) was a particular focus of the study as it had previously been found in higher concentrations in the liver and blood of older DR rats (Laganiere and Yu, 1989; Lang et al., 1989). In fact, MR regardless of how it has been imposed has shown a consistent reduction in oxidative stress across species (Zhang et al., 2022).

As methionine is the only precursor of cysteine in a cysteine-free diet, and cysteine is one of the three amino acids comprising glutathione, MR might logically be expected to reduce, rather than increase, glutathione levels. Yet despite that expectation, MR led to an *increase* in circulating glutathione throughout life (Richie et al., 1994). At 2 years of age, whole blood glutathione was dramatically -- 2.6-fold -- higher in the low methionine, compared to control, animals. However, glutathione levels in liver and kidney dropped substantially (60% reduction in liver, 25% reduction in kidney) consistent with a hypothesis that low levels of dietary methionine led to mobilization of liver and kidney glutathione stores. Oxidative damage to tissue macromolecules was not measured.

Several mouse studies of low methionine diets also showed increased longevity associated with lower body weight, but with some interesting differences from the rat experiments. For instance, in two studies using CB6F1 mice the semi-purified control diet contained only half as much methionine as the rat control diet even though both were based on the same AIN-76 diet (Miller et al., 2005; Sun et al., 2009). The first experiment using only females of this F1 hybrid stock, began like the earlier rat experiments feeding MR when the mice were only partially grown. Notably, about one-quarter of animals (but no controls) died by 6 months of age when fed very low methionine levels (0.1% and 0.12%), levels. Recall, such low levels of dietary methionine had been 100% fatal to rats within 30 days (Orentreich et al., 1993; Miller et al., 2005). Females of this mouse genotype were clearly more resistant than male rats to methionine deficiency. However from age 6 months when restriction was eased to 0.15% methionine, that is roughly 35% of the concentration in the control diet, restricted animals survived better than controls and exhibited slower development of eye lens opacity. Noteworthy is the fact that unlike the rats that ate less of the low methionine diet, these mice ate *more* of the low methionine diet, but still weighed significantly less than controls, demonstrating that the MR effect was not confounded by food restriction. The second experiment demonstrated that restriction did not have to be initiated in partially grown mice, as males of the same F1 genotype also lived longer on a low methionine diet when initiated a 1 year of age (Sun et al., 2009). This paradoxical result, where mice eat more but weigh less on a low methionine diet, was also seen in an independent study of male mice of a genetically heterogeneous background (Brown-Borg et al., 2014a), confirming this intriguing species difference of the impact of low methionine on food consumption.

Not all mouse studies of low methionine resulted in longer life though. A recent study using the same diets as previous mouse studies but an inbred (C57BL/6) background found no significant lengthening of life and only marginal impacts on health or frailty (Thyne and Salmon, 2023). Notably, this was the only study in which mice on a low methionine weighed more, not less, than controls. So genetic background of the mice appears important for the beneficial health and longevity effects of low methionine as it has for DR (Liao et al., 2010; Rikke et al., 2010; Unnikrishnan et al., 2021).

Interestingly, the longer-lived MR mice showed several phenotypes similar to long-lived genetic dwarf mice (Bartke et al., 2001; Hauck et al., 2001), including lower serum concentration of IGF-1, thyroxine, insulin, and glucose compared with controls. In addition, the low methionine mice showed greater resistance to acetaminophen-induced oxidative damage. A subsequent study using males of the same CB6F1 genotype with same low methionine (0.15%) diet initiated at age 1 year lived 7% longer than controls and had similar effects on IGF-1, thyroxine, insulin, glucose, and resistance to oxidative damage (Sun et al., 2009). That study demonstrated that low methionine could lengthen life even when initiated after mice had reached full adult growth.

The phenotypic similarities of MR and the long-lived dwarf mice made it particularly intriguing to examine how a low methionine diet would impact survival in dwarf mice themselves, particularly after it was discovered that genetic growth hormone disruption also altered methionine metabolism (Uthus and Brown-Borg, 2003). A nice series of survival experiments from the Brown-Borg lab using two dwarf mouse genotypes, one with disrupted GH production (Brown-Borg, 2016), the other with a disrupted GH receptor (Coschigano et al., 2003), fed low (0.16%), medium (0.43%, the standard diet), and high (1.3%) methionine diets from 8 weeks of age found that that the loss of GH activity also abolished the survival benefits of the low methionine diet (Brown-Borg et al., 2014b). Interestingly, the high methionine diet did not shorten survival relative to the medium (normal control) methionine diet, although it did increase the frequency of liver tumors. Together these studies suggest that altered methionine metabolism due to either dietary intervention or disruption of GH activity increases mouse longevity by similar, or at least partially-overlapping, mechanisms (Uthus and Brown-Borg, 2003; Uthus and Brown-Borg, 2006; Brown-Borg, 2009).

**FIGURE 1:**
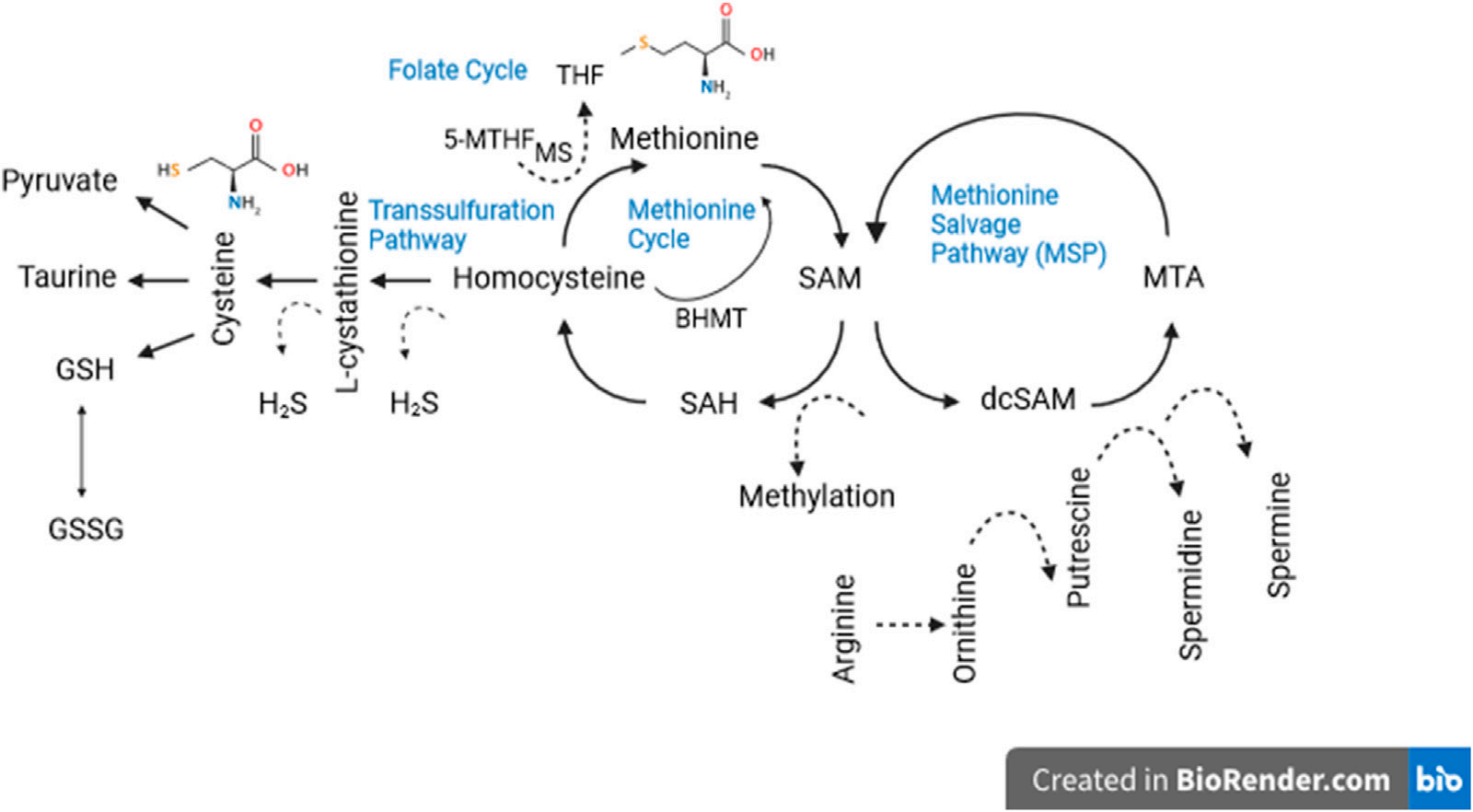
Methionine metabolism consists of a three part network: (1) the transsulfuration and (2) salvage pathways and (3) the methionine cycle itself. As can be seen, the methionine metabolic network consists of a host of aging-relevant intermediates and end-products. Dotted lines denote “side products” not directly involved in salvage or synthetic pathways. See text for details.

So far we have avoided mentioning some known side effects of a low methionine diet, except for growth retardation, but impaired reproduction (Lee et al., 2014; Reda et al., 2020) and low bone mineral density (Ables et al., 2012) are also common side effects. We have also avoided discussing the mechanism(s) by which low methionine diets may extend life and improve health. That is because by now it is obvious that these mechanisms are complex and only vaguely understood. Certainly they are multifactorial as the methionine metabolism network involves numerous intermediates and end-products involved in the biology of aging (Figure 1). A cursory summary of the processes hypothesized to be involved may be useful, however for a more complete discussion see Brown-Borg and Buffenstein (Brown-Borg and Buffenstein, 2017) or Babygirija and Lamming (Babygirija and Lamming, 2021). Dietary methionine is converted to SAM (S-adenosylmethionine), then to SAH (S-adenosylhomocysteine), then to homocysteine where it can be recycled to methionine or converted to cysteine via the transsulfuration pathway, which generates hydrogen sulfide (H_2_S) as by-product. H_2_S has been linked to virtually every hallmark of aging (Perridon et al., 2016). Cysteine, a nonessential amino acid, is a critical precursor for glutathione as mentioned earlier, but it is also a precursor of taurine, which was recently discovered to have multiple health and longevity benefits (Singh et al., 2023). Cysteine also plays a significant role in protein folding and stability via disulfide bonds between cysteine residues (Wiedemann et al., 2020). SAM is involved in methyl group transfers to all macromolecules but is particularly interesting for its impact on the epigenome which is increasingly appreciated to be centrally involved in aging via regulation or dysregulation of gene expression (Cole et al., 2017; Haws et al., 2020). Homocysteine is involved in immune function, blood clotting, and a host of aging-relevant processes.

It is important to re-emphasize that in the diets fed the vertebrate species discussed so far, contained low amounts of methionine and no cysteine. Therefore, there was no dietary source from which cysteine could be synthesized other than the methionine. However, certain microbes can synthesize cysteine and methionine from serine via the so-called reverse transsulfuration pathway. The extent to which microbial metabolism significantly affects methionine metabolism in vertebrates is unclear, but there is some evidence that it may be a significant source in invertebrates (Matthews et al., 2020).

#### Methionine (*Drosophila*)

The impact of varying dietary methionine availability has also been investigated extensively in *Drosophila* although several key differences between fly and laboratory rodent dietary intervention protocols are worth noting. Specifically, fly interventions are generally initiated only in adulthood (after eclosion) when growth is already largely determined, whereas a substantial number of rodent interventions were initiated when animals are only partially grown. Also, unlike rodent studies which for the past several decades did not include reproductive opportunities, fly studies often examine the impact of the intervention on reproduction (Bass et al., 2007; Grandison et al., 2009; Juricic et al., 2020). We consider this a substantial gap in understanding the translatability of laboratory rodent studies, as pregnancy and childbirth have significant effects on the subsequent physiology and health of women (La Vecchia et al., 1993; Vogel et al., 2024). Also, in flies, mated females eat more and are thus shorter-lived than virgin females (Carvalho et al., 2006).

Likely because fly dietary studies are initiated in adulthood and often include effects on reproduction, the composition of control diets have received considerably more attention, although no better standardization, than they have in rodent studies. One study revealed the special status of methionine among essential amino acids in flies. Using a control diet that maximized fecundity without compromising survival (Bass et al., 2007), and a 50% DR protocol which lengthened life and reduced fecundity, it was found that adding back essential amino acids consistently reduced longevity and increased fecundity–mimicking the fully-fed condition, in other words–with one exception. Adding back methionine restored fecundity without shortening lifespan (Grandison et al., 2009). These results do suggest that an appropriate ratio of methionine relative to other essential amino acids is critical to understanding the impact of dietary manipulation on longevity and fecundity.

A particularly thorough study along similar lines was performed by Lee, *et al.* (Lee et al., 2014), who evaluated a wide range of methionine concentrations under a wide range of dietary amino acid levels to assess both its deleterious and salubrious impact on survival and reproduction (cumulative eggs laid). These authors first established that relative to the lab’s standard diet, a 10-fold reduction in all amino acids increased longevity slightly but reduced fecundity by 3-fold. However, adding 1 mM of methionine to that low amino acid diet increased survival relative to the standard diet by greater than 80% while returning fecundity to the standard diet’s level. Adding more methionine shortened life and reduced fecundity of virgin and mated females but had no effect on males. Clearly the *ratio of methionine concentration* relative to other amino acids was critical to its beneficial or detrimental as well as sex-specific effects. Overexpression of genes inactivating mTOR signaling abolished these effects suggesting that the life extending effect of low methionine required inhibition but not elimination of mTOR signaling.

As mentioned earlier, reducing GH activity either via the hormone itself or through its receptor alters methionine metabolism in complex ways. Direct genetic manipulation of the methionine metabolic network has also had interesting impacts on longevity. For instance, using a series of inducible and tissue-specific transgenic constructs with a bacterially-derived enzyme -- methioninase, which degrades methionine to ammonia, α-ketobutyrate, and methanthiol -- to reduce *in vivo* methionine, Parkhitko and others found that they could both investigate the consequences of whole body and tissue-specific reduced methionine levels (Parkhitko et al., 2019). Using that approach, researchers observed that whole body, neuron-specific, muscle-specific, intestine-specific, and fat-body-specific reduction of methionine significantly extended life when flies were on a standard diet.

##### Branched-chain amino acids

A group of three other essential amino acids, the branched-chain amino acids (BCAAs) leucine, isoleucine, and valine, have also drawn interest due to their known impact on protein synthesis, food intake, and metabolic health (Le Couteur et al., 2020; Babygirija and Lamming, 2021). High plasma concentration of BCAAs are associated with obesity and insulin resistance in humans and laboratory rodents on a westernized diet supplemented with BCAAs develop obesity and insulin resistance (Newgard et al., 2009; Cummings et al., 2018). Also, BCAAs, in particular leucine, are known to activate mTOR, which multiple studies in multiple species has been shown to shorten life and compromise health (Vellai et al., 2003; Kapahi and Zid, 2004; Ravikumar et al., 2004; Kaeberlein et al., 2005; Harrison et al., 2009; Dazert and Hall, 2011).

A particularly thorough fly study of the impact of a diet restricting all thee BCAAs by 85% compared to a diet similarly reduced in three other essential amino acids (threonine, histidine, lysine) found that both diets increased food intake and yet lengthened life to about the same extent. I should note that the control diet in this case was unique such that each essential amino acid was equally limiting to growth and reproduction (Juricic et al., 2020). In this study, nonreduced amino acids were proportionally increased so that all diets had the same amino acid concentrations. Thus in this context, restricting BCAAs had the same effect as restricting three other essential amino acids.

In virgin laboratory mice interactions among essential amino acids have been reported to be important in understanding the effects of varying BCAA levels. Specifically doubling the levels of BCAAs in a fixed 18% protein diet led to increased food intake and reduced longevity compared with control or restricted BCAA diets. In the same study, low BCAA diets (50% or 20% of controls) did not lengthen life (Solon-Biet et al., 2019). The hyperphagia (and presumably the life-shortening) effect of the high BCAA turned out not to be due to increased BCAA levels themselves but to the imbalance between BCAAs and two other essential amino acids, threonine and tryptophan. When levels of these two amino acids were elevated in the high BCAA diet to balance the BCAA overabundance, the hyperphagia disappeared.

As previously noted, life-long protein restriction typically increases longevity, reduces body weight, and increases food intake in virgin laboratory mice. A study that initiated restricting BCAAs by two-thirds at age 16 months while keeping dietary protein levels constant at 18% improved aspects of health but did not increase longevity in either sex of C57BL/6 mice from 23 months of age (Richardson et al., 2021). However, when the same diet was initiated in the same mouse strain at weaning and continued throughout life, there was little impact on females but males showed substantially increased median (∼30%) and maximum (∼12%) longevity. This represents one more example of the increasingly common observation that life- and health-extending diets or drugs can benefit one sex but have no effect on the other.

Among the BCAAs, special attention has fallen on isoleucine due to its unique impact on metabolic health in virgin laboratory mice (Yu et al., 2021). Indeed, reducing isoleucine by two-thirds in a 21% protein diet was seen to improve multiple aspects of metabolic health and increase longevity substantially (33% median) in males and more modestly (7% median) in females relative to both a control diet and to a diet in which all amino acids were reduced by two-thirds (Green et al., 2023). The dietary manipulations were initiated at 6 months of age in genetically heterogeneous mice. Interestingly, in this study the low amino acid diet–which presumably is equivalent to a reduced protein diet, did not have the same metabolic benefits nor did it lengthen the life of either sex. A more recent investigation using C57BL/6 mice found that both reduced amino acid and reduced isoleucine diets -- the same diets as employed in the previously-mentioned study -- conferred multiple health benefits even when initiated in older mice (20 months of age) (Yeh et al., 2023). In an interesting complementary study, transient 5–7 days of complete isoleucine deprivation early in life leads to a small (∼5%) increase in fly longevity (Fulton et al., 2024).

In the previous several paragraphs, we have emphasized that virgin laboratory mice were used, as is virtually always the case in modern mouse studies. There are two reasons we feel that pointing out the reproductive and laboratory status of these standard mouse studies may be important. First as noted previously, there is a gap in our knowledge about virtually all dietary manipulations in mice that have mated and/or reproduced. Taking one recent example of how this could be relevant, a provocative study appeared recently that investigated the impact of 30% calorie restriction, as well 68% amino acid and 68% BCAA reduction on ovarian reserve in young female C57BL/6 mice, the same strain used in many of the previously described mouse studies (Veiga et al., 2024). In the reduced BCAA group, total amino acid levels were maintained by proportionally increasing all of the other amino acids. Recall that one of the surprising findings with DR (and reduced tryptophan) was the ability of animals to reproduce at ages when *ad lib* fed animals were long postreproductive. The interesting finding from this small study was that after 3.5 months on these diets, the calorie and amino acid restricted groups showed preserved ovarian reserve, whereas the reduced BCAA group did not. If confirmed in humans, this result may have translational significance. Women, especially college-educated women, are postponing reproduction more and more often which is associated with declining fertility rates in developed countries (Beaujouan, 2020). The possibility that diet, especially diets with putative health-extending effects, could affect later fertility is worth understanding more about.

We emphasized *laboratory* mice because for the past several decades, laboratory mice have generally been reared and maintained in specific pathogen free (SPF) conditions. There are some good reasons for doing so having to do with lab-to-lab experimental reproducibility that SPF conditions have become the norm in biomedical research (Baker, 1998; Miller and Nadon, 2000). However, protecting laboratory animals from specific pathogens also prevents them from normal microbiological exposure (Beura et al., 2016). Lack of a normal microbiological experience has dramatic effects on the laboratory mouse immune system, something thought to be one factor in the poor translatability of mouse studies to human medical biology (Beura et al., 2016; Abolins et al., 2017; Rosshart et al., 2019). BCAAs are known to be important in immune function and BCAA reduction in laboratory rodents has been reported to inhibit several aspects of immune function as well as increase susceptibility to pathogens (Tsukishiro et al., 2000; Calder, 2006). For these reasons, the remarkable promise of low protein or reduced amino acids diets warrants investigation under conditions that are more relevant to the lived human experience than our current standard laboratory practice.

## Conclusion

The impact of diet on health is complex. Various diets, from low calorie to low protein to low methionine, BCAA, or isoleucine formulations, have shown that dietary modulation can affect later life health in laboratory species. Whether these dietary enhancements of later life health will be translatable to humans is a question begging to be answered.

So far surveys of humans on plant-based low methionine or low sulfur amino acid (methionine + cysteine) diets have been reported to be associated with several beneficial health outcomes such as lower cardiometabolic risk factors (Dong et al., 2020) or diabetes-related mortality (Dong et al., 2022). Short-term (4–12 weeks) clinical trials indicate that low sulfur amino acid diets as in most animal studies lead to weight loss, lower total and LDL cholesterol, and other salubrious changes (Richie et al., 2023; Olsen et al., 2024). The cancer field has been particularly interested in low methionine diets as both cell-based and preclinical studies confirm that cancer cells hunger for methionine (Hoffman et al., 2019b). Yet short-term trials of medical methionine restriction, especially when combined other cancer therapies, have been generally less than successful largely because of low palatability of the diet (Hoffman, 2019; Mattes et al., 2024). Clearly if low methionine or low sulfur amino acid diets are to be sustainable, the plant-based approach is more likely to be successful.

It is time to determine in human studies whether these low these amino acid restricting diets unlike chronic DR, are sustainable over the long-term and what the long-term health consequences might be. It is also important to discover how the diets affect mood, energy, and interact with other healthy-enhancing lifestyle or pharmaceutical interventions such as exercise or geroprotective drugs (Elliehausen et al., 2023). We have reached the “translation stage” of biological aging research. It will be curious to see how successful that translation will be.
